# Heavy Metal and Metalloid Pollution of Soil, Water and Foods in Bangladesh: A Critical Review

**DOI:** 10.3390/ijerph15122825

**Published:** 2018-12-11

**Authors:** M. Mominul Islam, Md. Rezaul Karim, Xin Zheng, Xiaofang Li

**Affiliations:** 1 Key Laboratory for Agricultural Water Resources, Center for Agricultural Resources Research, Institute of Genetics and Developmental Biology, Chinese Academy of Sciences, Shijiazhuang 050021, China; momin.anft@gmail.com (M.M.I.); zhengxin@sjziam.ac.cn (X.Z.); 2 University of Chinese Academy of Sciences, Beijing 100049, China; 3 Department of Applied Nutrition and Food Technology, Islamic University, Kushtia 7003, Bangladesh; 4 CMLR, Sustainable Minerals Institute, The University of Queensland, Brisbane, Queensland 4072, Australia

**Keywords:** heavy metals, metalloids, pollution, industrialization, food, toxicity

## Abstract

Bangladesh is a densely populated developing country. Both industrialization and geological sources have caused widespread heavy metal and metalloid pollution in Bangladesh, which is now posing substantial threats to the local people. In this review, we carried out one of the most exhaustive literature analyses on the current status of Bangladesh heavy metal and metalloid pollution, covering water, soil, and foods. Analysis showed that soils near high traffic and industrial areas contain high concentrations of heavy metals and metalloids. Agricultural land and vegetables in sewage-irrigated areas were also found to be heavy metal- and metalloid-contaminated. River water, sediment, and fish from the Buriganga, Turag, Shitalakhya, and Karnaphuli rivers are highly contaminated with cadmium (Cd), lead (Pb), and chromium (Cr). Particularly, groundwater arsenic (As) pollution associated with high geological background levels in Bangladesh is well reported and is hitherto the largest mass poisoning in the world. Overall, the contamination levels of heavy metals and metalloids vary among the cities, with industrial areas being most polluted. In all, this review provides a quantitative identification of the As, Pb, Cd, and Cr contamination hotspots in Bangladesh based on the literature, which may be useful to environmental restorationists and local policy makers.

## 1. Introduction

Heavy metals and metalloids are non-biodegradable in nature and can affect human health directly and indirectly [1]. Chronic exposure of heavy metals and metalloids can damage various organs like kidneys, liver, lung, brain, and bones [2,3]. Bangladesh is one of the most densely populated countries in the world with a population density of 1278 people per square kilometer [4]. Case reports on poisoning of heavy metal and metalloid exposure have been increasing in recent years in Bangladesh. In Bangladesh, ground water arsenic (As) contamination has become a major public health problem. Millions of people are drinking As-contaminated water and this mass poisoning is the biggest As disaster in the world [5,6,7]. Rapid industrialization, urbanization, and various anthropological activities also have driven the wide dispersion of cadmium (Cd), lead (Pb), and chromium (Cr) in the environment. Rivers surrounding Dhaka and Chittagong such, as the Buriganga, Turag, Shitalakhya, and Karnaphuli rivers are highly polluted by Cd, Pb, and Cr [8,9,10,11]. Industrial effluents and sewage can deteriorate river water in many aspects. Fish species from polluted rivers also contain elevated concentrations of heavy metals [12,13]. Soil near the industrial areas of the big cities in Bangladesh, such as Dhaka, Gazipur, Chittagong, and Bogra, displayed excess heavy metals and metalloids [14]. High traffic loads [15] are also responsible for high heavy metal and metalloid pollution in water and soil. Meanwhile, agricultural products from contaminated soil are frequently found to contain high concentrations of heavy metals and metalloids, which may impact human health profoundly [16].

**Figure 1:**
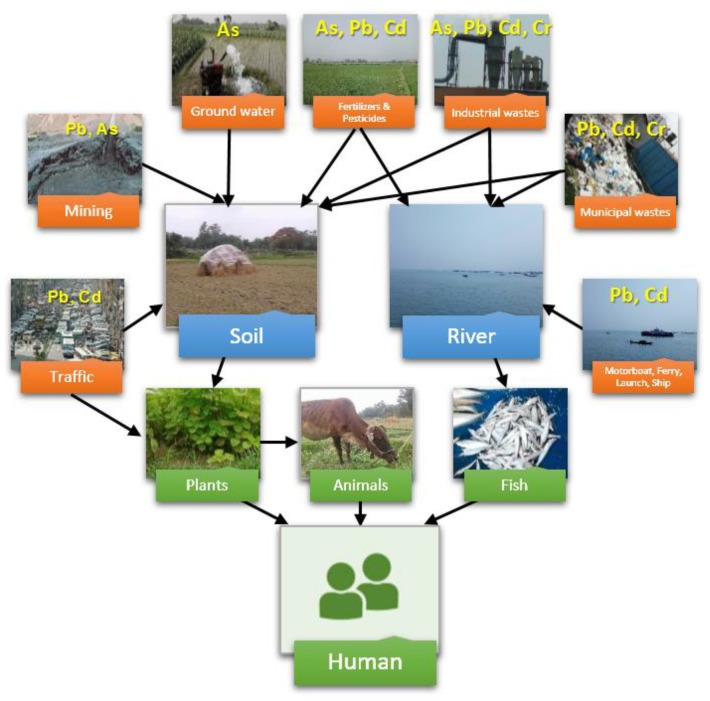
Major pathways of heavy metal and metalloid dispersion and human exposure in Bangladesh.

The widespread heavy metal and metalloid pollution in Bangladesh has received attention worldwide, and there have been several excellent reviews dedicated to specific metals or environmental media [6,17,18]. This paper aimed to conduct an extensive literature review in order to systematically evaluate the heavy metal and metalloid pollution status of heavy metals and metalloids in Bangladesh in recent decades. Metadata was collected from government reports and publications covering As, Pb, Cd, and Cr concentrations in soil, river, and crops. Based on this data, major pathways for the exposure of local people to metals and metalloids were depicted and hotspot regions for risk management were located (Figure 1), which may provide useful information to government and environmental researchers.

## 2. Soil Heavy Metal and Metalloid Pollution

Major sources of soil heavy metal and metalloid pollution include municipal wastes, industrial effluents, chemical fertilizers, and pesticides [19]. Irrigation with contaminated groundwater and river water are also responsible for soil contamination. Heavy metal and metalloid pollution of farmland and crops can substantially impact food safety as well as human health [20]. Soils in Bangladesh polluted by heavy metals and metalloids have been found to be impacted by various pollution sources (Table 1).

In Bangladesh, cultivation in the dry season mostly depends on irrigation by deep shallow tube wells (STWs). Bangladesh has the highest percentage of As-contaminated STWs, and yearly increases of up to 0.1 mg of As per kg of soil can occur as a result of irrigation, especially in paddy fields [21]. Duxbury et al. [22] stated that paddy fields irrigated with As-contaminated water for ten years would add 5–10 mg/kg As into soil. Agricultural soil irrigated with Shitalakhya river water in Narayangonj presents elevated Pb (28.13 mg/kg), Cd (0.97 mg/kg), and Cr (69.75 mg/kg), which are higher than safe limits [23]. Rice is the staple food in Bangladesh, with average rice consumption of 400 to 600 g per day by an adult [24]. Therefore, risks from inorganic As in rice from regions of high soil As pollution may affect local people directly [24,25,26].

Industrial wastes and chemical pesticides have also contributed to soil As contamination in Bangladesh. In urban areas, untreated effluents from industries are directly adding heavy metals and metalloids into the nearby water and soil [14]. A number of studies on farmland nearby the Dhaka Export Processing Zone (DEPZ) indicated that irrigation with contaminated sewage water increased soil heavy metal and metalloid load [14,40]. Hasnine et al. [40] stated that agricultural fields nearby the DEPZ displayed Cr concentrations of 2753.2 mg/kg in the surface soil and 1039.2 mg/kg in the sub-surface layer. Results by Rahman et al. [14] showed that in the dry season agricultural soil nearby the DEPZ contained 4043 mg/kg of As and 49.66 mg/kg of Cr. Waste water from the Hazaribagh leather industrial area in Dhaka was found to be responsible for high Cr (976 ± 153 mg/kg) concentrations in the local soil [27]. Soils from several industrial areas in Gazipur and Barisal also presented much higher Cd than the recommended values [29,32].

Mining has a great impact on soil heavy metal and metalloid load in some parts of Bangladesh. Coal, coal ash, and coal- fired boilers have great impacts on environmental Pb. Soil from the coal mine affected farmland at Barapukuria, Dinajpur, was shown to contain excess Pb at a level of 433 ± 5.66 mg/kg [36]. Some other important sources for soil heavy metals and metalloids pollution in Bangladesh have been reported as well. Industrial and urban effluents release large quantities of heavy metals and metalloids, which are responsible for high heavy metals and metalloids in soil and water. Soil from Chittagong and Bogra city were found to be polluted by Cd mainly due to rapid industrialization and urbanization in recent decades [30,31]. Excessive use of phosphate fertilizers and pesticides are responsible for increasing heavy metals and metalloids in the soils of commercial and residential vegetable plots in Pakshi, Pabna [37].

## 3. Water Heavy Metal and Metalloid Pollution

**Figure 2:**
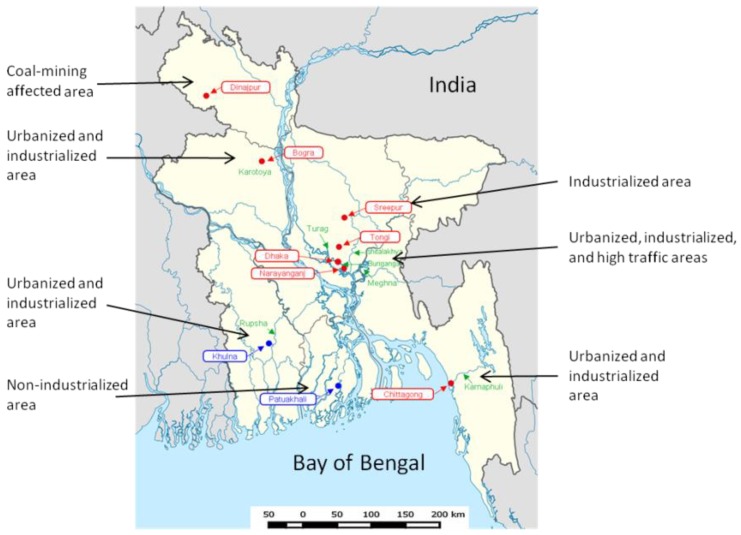
Pb, Cd, and Cr pollution hotspots in Bangladesh. Hot spots of heavy metal pollution were in red. As is not considered here.

Most areas of Bangladesh are rainy regions that are rich in rivers. The river systems and rainfall provide an important way for the regional and cross-regional dispersal of pollutants, particularly heavy metals and metalloids [41] (Figure 2). The heavy metal and metalloid water pollution in Bangladesh has been well documented in recent years.

Arsenic pollution in Bangladesh is one of the well-studied environmental issues in the world. Arsenic is widespread in the Earth’s crust [42]. In Bangladesh, drinking water is one of the major sources of inorganic As because of geological factors, especially in the Ganga-Brahmaputra-Meghna river basin [6,43]. The permissible level of As in drinking water established by the World Health Organization (WHO) and the United States Environmental Protection Agency (USEPA) is 10 µg/L, however, in many developing countries like Bangladesh it has been adjusted to 50 µg/L because of inadequate analytical instruments for lower arsenic concentrations in water [44,45]. In water, As was found mostly in the oxidation states (+III and +V) [46]. In the 1970s and 1980s, the Bangladesh government and United Nations International Children’s Emergency Fund (UNICEF) set up millions of hand tube wells around the country to combat against water- and foodborne communicable diseases. Unfortunately, these hand tube wells became major sources of As [7,47]. The Department of Public Health Engineering (DPHE) of Bangladesh first surveyed groundwater As contamination in 1993 [47,48]. In Bangladesh, 61 districts (excluding the Hill tracks areas) out of 64 are affected by As, and the level of As in drinking water is more than 50 µg/L [47,48]. About 20 million people in Bangladesh are using such tube wells water with excess As [7]. Northwest regions of Bangladesh are more affected by As [6,49]. According to the Bangladesh Bureau of Statistics (BBS), about 77 million Bangladeshi people are affected by As-contaminated water [50]. The As crisis in Bangladesh was thought to be the largest mass poisoning in human history [44]. In recent years, new cases of toxicity have continued to emerge in different parts of the country [44,49]. During 1996–2006, many government, national, and international organizations, including educational institutions, set up As monitoring and mitigation programs in Bangladesh [47,51,52]. Marking of the contaminated tube wells was one of the major steps taken by the mitigation program to identify the contaminated wells, however, this has had a limited effect at lessening the calamity [53]. Now, one of the most important mitigation programs has been setting up As-free deep tube wells in the most contaminated areas in order to provide safe drinking water to the local people.

Bangladesh is a riverine country, and rivers have a great impact on its transportation, fisheries, and industrial activities. The biodiversity and ecology of rivers can be substantially affected by metal and metalloid contamination [54]. Untreated and partially treated effluents from industries are the main cause of elevated heavy metals and metalloids in river water [55]. The water of the Buriganga River in the Hazaribagh area receives daily about 22,000 L of toxic wastewater from 200 tanneries [56]. Frequent irrigation with this river water can contaminate agricultural soil and ultimately affect crop yield as well as food safety [57,58].

Dhaka is the largest city in Bangladesh, located on the bank of the Buriganga River. The other important rivers near Dhaka are the Turag, Balu, Dhaleswari, and Shitalakhya rivers. During the last few years heavy metal and metalloid load together with organic pollutants of these rivers increased to unexpected levels from various sources; therefore, these rivers are known as the “Biologically Dead Rivers” in Bangladesh [59]. The sediments of the Buriganga River also contain concentrations of Pb, Cd, and Cr higher than the standard values [60,61] (Table 2). The Turag River contains Pb (0.073–0.1mg/L) and Cr (0.039–0.061 mg/L) in higher concentrations because of the heavy industrialization on both sides of this river [55]. The sediment of this river also contains Cd (0.8 mg/kg) and Cr (178 mg/kg) in excess concentrations [62]. More case reports on river sediment pollution by heavy metals and metalloids can be found in Table 3.

The heavy metal and metalloid pollution of Bangladesh rivers was also reflected by the many case reports on heavy metal and metalloid pollution in fish in recent years (Table 4). A variety of fish species from the Buriganga river were found to contain Pb, Cd, and Cr concentrations above the food safety guidelines by the World Heath Organization and Food and Agriculture Organization [12,63]. For example, *Labeorohita* (Rohu) from the Buriganga River was determined to be polluted by Pb (6.98 mg/kg) and Cr (18.84 mg/kg) [12].

The Shitalakhya River is located on the northwestern side of the capital. Sediments from the Shitalakhya River were mainly polluted by As (14.02 mg/kg) and Cr (74.82 mg/kg) [64].

The largest port of the country is situated at the bank of the Karnaphuli River. This river is contaminated by various industrial wastes and shipping vehicles [79]. Ali et al. [8] stated that the sediment of this river contained excess Cd and Cr. Islam et al. [13] found that Chapila fish from this river was highly contaminated by Pb (4.94 ± 0.60 mg/kg). The sediments of coastal ship breaking areas in Chittagong, such as the Bhatiari and Sonaichhari areas, were substantially contaminated by Pb and Cd [80].

The water of the Karotoa River is polluted by various industrial, pharmaceutical, and municipal wastes from the Bogra city and the sediment of this river is severely contaminated by Cd (10.85 mg/kg) [73]. It was found that fishes from the Meghna River and the Paira River were both contaminated by Pb [74,77]. Fortunately, river water, sediments, and fishes from non-industrial areas like Rupsha [81,82] in Khulna, Possur [69], near the Mongla port, Bramaputra [33], near Chilmari, and Kurigram and Dakatia [83], near Chandpur, remain uncontaminated based on available reports.

## 4. Crop Heavy Metal and Metalloid Pollution

As a tropical country, Bangladesh produces more than 90 kinds of vegetables and 60 kinds of fruits [84]. Environmental pollution and nature of the soil directly affect the heavy metal and metalloid content in foods. Chemical pesticides and fertilizers containing heavy metals and metalloids are both major sources of heavy metals and metalloids in foods. Some trace metals are essential in plant nutrition; however, excess heavy metals and metalloids can accumulate in various edible and non-edible parts of plants [85]. Basically, leafy vegetables are more liable to heavy metal and metalloid contamination, due to their rapid growth and direct transfer of metals and metalloids to the leafy parts [86].

Irrigation with As-contaminated ground water is the primary cause of food As contamination in Bangladesh. Organic As in foods is considered to be less harmful. However, As-contaminated crops may contain a large portion of inorganic As [87,88]. Besides drinking water, food As exposure was also found to an be important pathway responsible for As poisoning [21,84,89,90]. Alam et al. [84] found that vegetables grown in the Samta village were contaminated by As. Rice from Brahmanbaria also was observed to contain As (0.24 mg/kg) and Cd (0.331 mg/kg) in higher concentrations than the established safe limits [90]. Safe limits for main metals and metalloids in food stuffs are as follows: As 0.1 mg/kg; Pb 0.05 mg/kg; Cd 0.05 mg/kg; and Cr 2.3 mg/kg [91].

Various studies showed that plants grown nearby industrial areas retain more heavy metals and metalloids than those from non-industrial areas (Table 5). Cabbage (*Brassica oleracea*) from agricultural land nearby DEPZ contains Pb (22.09 mg/kg), Cd (2.05 mg/kg), and Cr (7.58m mg/kg) in higher concentrations than the safe limits [92]. Edible parts of Spinach (*Spinacia oleracea*) from the Hazaribagh leather industrial area of Dhaka presented higher levels of As (0.26 ± 0.22 mg/kg), Pb (11.48 ± 4.98 mg/kg), Cd (0.32 ± 0.094 mg/kg), and Cr (44.48 ± 12.59 mg/kg) [27]. Bottle gourd (*Lagenaria siceraria*) (Pb 1.16 ± 0.01 mg/kg) and water spinach (*Ipomoea aquatica*) Cr (3.21 ± 0.023 mg/kg) from the Vatiary industrial area of Chittagong both exceeded the safe limits [93]. Potato (*Solanum tuberosum*) from Bogra was found to be polluted by Pb and Cd [94].

Vegetables grown in high traffic areas were also found to contain higher concentrations of heavy metals and metalloids. Naser et al. [95] found that pumpkin (*Cucurbita maxima*) grown close to the highway in Joydevpur, Gazipur, contained Pb (4.76 ± 1.03 mg/kg) and Cd (0.20 ± 0.02 mg/kg) in concentrations much higher than those grown in distant areas.

Irrigation with contaminated river water may substantially affect the metal and metalloid concentrations of vegetables. Red amaranth (*Amaranthus cruentus*) collected from agricultural land surrounding the Turag River were considerably polluted by Pb (1.99 ± 0.44 mg/kg) and Cd (0.84 ± 0.17 mg/kg) [96]. Purple amaranth (*Amaranthus lividus)* from agricultural land surrounding the Shitalakhya river was polluted by Pb and Cd as well [23].

Market samples provide important insights into the average contamination levels of heavy metals and metalloids in foods in Bangladesh. Rice, fish, and vegetables from Kawran Bazar, Dhaka, were all found to contain Cd and Pb in higher concentrations than the safe limits [97]. In their market-based study, Shaheen et al. [98] showed that mangos (*Mangifera indica*) presented excess Pb and tomatoes (*Solanum lycopersicum*) contained excess Cd.

## 5. Conclusions

Heavy metal and metalloid contamination from both geological and industrial sources has become a major issue for the people of Bangladesh in recent years. Results in the literature clearly showed that heavy metal and metalloid risks in Bangladesh are associated mainly with mining, industrialization, and urbanization. Dense river systems allow the heavy metals and metalloids to be dispersed more easily in some parts of Bangladesh. This review provides one of the most exhaustive literature reviews on the heavy metal and metalloid pollution status in Bangladesh and indicates the urgent need for all relevant sectors to control the emission of heavy metals in Bangladesh.

## Figures and Tables

**Table 1:** Recent reports on heavy metals and metalloids pollution in Bangladesh soil (mg/kg).

City	Sampling Site	Sampling Time/Site Number	As	Pb	Cd	Cr	Reference
Dhaka (Hazaribagh)	Leather industrial area	-/4	1.94 ± 0.39	50.32 ± 4.36	0.45 ± 0.11	976 ± 153	[27]
Dhaka (City area)	High traffic areas	-/20	NA	45.68 ± 25.5	0.38 ± 0.14	31.75 ± 17.55	[28]
Dhaka (DEPZ), Dry Season	Farm land surrounding industrial area	February 2010 to April 2011/20	4073.1 ± 1116	27.6 ± 7.9	0.0072 ± 0.02	49.66 ± 34.7	[14]
Dhaka (DEPZ), Wet Season	Firm land surrounding industrial area	February 2010 to April 2011/20	2326.2 ± 3274	9.61 ± 11.3	1.04 ± 2.03	34.2 ± 26.5	[14]
Gazipur (City area)	High industrial and traffic areas	-/3	NA	27.95	0.41	29.21	[29]
Bogra (City area)	Urban and industrial areas	October to November 2010/14	NA	9.61 ± 7.483	6.95 ± 0.95	4.05 ± 2.03	[30]
Chittagong (City area)	Industrial and high traffic areas	-/21	NA	7.33 ± 0.40	2.43 ± 0.17	NA	[31]
Barisal	Surrounding cement industry	-/4	2.13 (1.45–2.5)	23.39 (11.6–38.52)	0.62 (0.5–0.77)	38.26 (22.05–55.0)	[32]
Barisal	Surrounding textile industry	-/4	1.41(1.36–1.45)	18.48 (8.2–33.22)	1.9 (0.9–3.2)	132.5 (95.1–185.4)	[32]
Barisal	Surrounding medicine industry	-/4	1.67(1.05–1.77)	11.42(10.6–12.68)	0.78 (0.5–0.87)	25.73 (15–30)	[32]
Kurigram (Chilmari)	Bank of Brahmaputra river	March 2012/15	NA	26.7	0.48	34.7	[33]
Tangail (Tarutia)	Industrial area	March–April 2016/15	6.11	17.46	2.01	11.56	[34]
Dinajpur (Barapukuria)	Mine affected paddy field soil	December 2009/10	22.44	188.61	NA	NA	[35]
Dinajpur (Barapukuria)	Mine affected farmland soil	30	17.55 ± 5.66	433 ± 95	NA	NA	[36]
Pabna (Pakshi)	Commercial and residential areas	6	4200 ± 16.80	21.29 ± 0.47	<0.1	28.194 ± 0.17	[37]
Standards			0.11	200	0.48	11	[38]
Standards for industrial wastes (Irrigated land) mg/L			0.2	0.1	0.05	1.0	[39]

Notes: NA, not applied/reported; DFPZ: Dhaka Export Processing Zone. For metals and metalloids concentrations, some values were reported with standard errors, and some were reported with the concentration range in brackets.

**Table 2:** Heavy metals and metalloids pollution in major Bangladesh rivers (mg/L).

City	River	Major Sampling Location	Sampling Period	As	Pb	Cd	Cr	Reference
Dhaka	Buriganga	Kawtail, Postagola, Sodorghat, Modinanagar	October 2012 to August 2013	NA	NA	0.0104 ± 0.006	0.177 ± 0.11	[65]
Dhaka	Buriganga	Both sides of River from Rayer Bazaar to Pagla (30 km)	March 2010	0.134 (0.005–0.22)	0.119 (0.1–0.21)	0.059 (0.03–0.09)	0.114 (0.012–0.18)	[66]
Dhaka	Buriganga	Balughat, Shawaryghat, Foridabad		NA	0.065 ± 0.0047	0.0093 ± 0.0014	0.587 ± 0.0441	[63]
Dhaka	Turag	Tongi Heavy Industrial Area	NA	NA	0.073–0.1	0.002–0.003	0.039–0.061	[55]
Dhaka	Tongi (lake)	Tongi Heavy Industrial Area	March–April 2008	0.002	0.002	0.003	NA	[67]
Chitagong	Karnaphuli	Fishery ghat, Chaktikhal, Mojjartek, Kalurghat	NA	NA	0.14 ± 0.031	0.01 ± 0.002	0.25 ± 0.068	[13]
Chitagong	Karnaphuli	Fishery Ghat, Sea port, Custom House, marine Academy Bangladesh Jetty	September 2014 (summer)	0.023 ± 0.007	0.0098 ± 0.0047	0.0065 ± 0.003	0.067 ± 0.017	[8]
Chitagong	Karnaphuli	Fishery Ghat, Sea port, Custom House, marine Academy Bangladesh Jetty	March 2015 (winter)	0.034 ± 0.0098	0.0168 ± 0.0061	0.0106 ± 0.0045	0.087 ± 0.0174	[8]
Bogra	Karatoa	Bogra district urbanized area	February–September 2013 (winter)	0.046 ± 0.027	0.035 ± 0.019	0.011 ± 0.008	0.083 ± 0.027	[68]
Bogra	Karatoa	Bogra district urbanized area	February–September 2013 (summer)	0.037 ± 0.024	0.027 ± 0.015	0.008 ± 0.006	0.073 ± 0.027	[68]
Bagerhat	Pasur	Near Mongla port	January to June 2013	NA	NA	NA	0.02	[69]
Mongla, Bagerhat	Pasur	NA	NA	0.0276–0.01673	0.01269–0.04267	0.0042–0.0198	0.0276–0.07739	[70]
Kurigram	Brahmaputra	Chilmari	NA	NA	0.037	NA	NA	[33]
Standards for Irrigation (maximum concentration)				0.1	0.01	5.0	0.1	[71]

Notes: NA, not applied/reported. For metal and metalloid concentrations, some values were reported with standard errors, and some were reported with concentration range in brackets.

**Table 3:** Heavy metal and metalloid pollution in river sediments (mg/kg).

River	City/Sampling Site	Sampling Period/Number of Sampling Site	As	Pb	Cd	Cr	Reference
Buriganga	Dhaka (Hazaribagh to Meherbagh)	January 2014/7	NA	31.4	1.5	173.4	[60]
Buriganga	Dhaka (Kholamura launch terminal to Postogola Bridge)	Summer 2009/20	14	475	4.7	511	[60]
Buriganga	Dhaka	Winter 2009/20	16	478	5.9	709	[72]
Buriganga	Dhaka (Watchpur Ghat to Badamtoli Ghat)	-/5	NA	79.8 ± 16.9	0.8 ± 0.55	101.2 ± 42.2	[61]
Buriganga	Dhaka (Balughat, Shawaryghat and Foridabad)	-/3	NA	69.75 ± 4.13	3.33 ± 0.77	177.53 ± 30.19	[63]
Turag	Tongi Bridge to Ashulia	-/15	NA	18.3	0.8	178	[62]
Turag	Tongi Bridge, to Taltola Bridge	NA	NA	32.78 ± 3.32	0.28 ± 0.33	43.02 ± 18.31	[9]
Karnaphuli	Chittagong (Fishery Ghat, Sea port, Custom House, Marine Academy Bangladesh Jetty)	September 2014 (summer)/7	16.79 ± 4.70	38.33 ± 12.74	1.51 ± 0.64	70.06 ± 30.93	[8]
Karnaphuli	Chittagong (Fishery Ghat, Sea port, Custom House, Marine Academy Bangladesh Jetty)	March 2015 (winter)/7	23.81 ± 6.39	49.04 ± 15.06	2.50 ± 0.85	92.11 ± 33.16	[8]
Karnaphuli	Chittagong (Fishery Ghat, Chaktikhal, Mojjartek, Kalurghat)	February to April during 2013/5	NA	4.96 ± 0.60	0.24 ± 0.02	0.76 ± 0.12	[13]
Karatoa	Bogra (Bogra district urbanized area)	February–September 2013 (winter)/8	27 ± 17	63 ± 16	1.5 ± 0.77	118 ± 50	[68]
Karatoa	Bogra (Bogra district urbanized area)	February–September 2013 (summer)/8	22 ± 16	54 ± 15	1.0 ± 0.53	99 ± 38	[68]
Karatoa	Bogra (Bogra City area)	March,2011/5	NA	69.81 ± 27.57	10.86 ± 0.92	8.37 ± 3.35	[73]
Pasur	Mongla port in the Sundarbans mangroves	January to June 2013/3	NA	6.919	NA	19.369	[69]
Pasur	Mongla	NA	3.15–19.97	7.34–55.32	0.39–3.17	20.67–83.70	[70]
Paira	Patuakhali	February–March and August–September 2012/8	19 ± 3.0	49 ± 11	1.2 ± 0.73	67 ± 27	[74]
Standards			4.8	17	0.09	92	[75]
Standards			NA	31	0.6	26	[76]

Notes: NA, not applicable/reported. For metal and metalloid concentrations, some values were reported with standard errors, and some were reported with the concentration range in brackets.

**Table 4:** Heavy metal and metalloid pollution of fishes from Bangladesh rivers (mg/kg).

City	River	Sampling Time	Sample site	Species/Local name	As	Pb	Cd	Cr	Reference
Dhaka	Buriganga	August to September 2013	Kamrangir Char and Amin Bazar	*Puntius ticto*	0.32 ± 0.01	3.05 ± 0.09	0.02 ± 0.00	5.54 ± 1.52	[12]
				*Puntius sophore*	0.19 ± 0.01	3.16 ± 0.08	0.02 ± 0.00	4.33 ± 1.35	
				*Puntius chola*	0.17 ± 0.00	2.32 ± 0.08	0.01 ± 0.00	3.57 ± 1.60	
				*Labeo rohita*	0.73 ± 0.03	6.98 ± 0.23	0.04 ± 0.00	18.84 ± 1.72	
				*Glossogobius giuris*	0.20 ± 0.01	1.77 ± 0.10	0.01 ± 0.00	5.13 ± 0.96	
Dhaka	Buriganga	Pre-monsoon period	Balughat, Shawaryghat and Foridabad	*Gudusia chapra* (chapila)	NA	9.12	0.83	6.27	[63]
				*Glossogobius giuris* (baila)	NA	9.58	0.81	6.13	
				*Cirrhinus reba* (tatkeni)	NA	8.03	0.76	6.75	
				*Channa punctatus* (taki)	NA	10.31	0.86	5.73	
				*Mystus vittatus* (tengra)	NA	12.32	1.09	5.47	
				*Pseudeutropius atherinoides*	NA	8.95	0.95	7.34	
Chittagong	Karnaphuli	February to April during 2013	Fishery ghat, Chaktikhal, Mojjartek, Kalurghateast zone and Kalurghat west zone	Poua	NA	0.886	0.066	0.569	[13]
				Chring	NA	1.843	0.744	1.077	
				Rita	NA	2.861	0.179	0.064	
				Chapila	NA	7.707	0.483	0.099	
Narayangong	Meghna	January 2016 (winter season)	Effluent discharge area	*Tanualosa ilisha*	NA	0.67	0.092	0.05	[77]
				*Colisa chuna*	NA	0.11	NA	1.6	
				*Labeo calbasu*	NA	1.91	NA	1.12	
				*Labeo rohita*	NA	NA	0.04	0.57	
				*Stinging catfish*	NA	1.56	NA	3.01	
				*Colisa lalia*	NA	6.75	NA	NA	
Potuakhali	Paira	February–March and August−September 2012	NA	*Cyprinus carpio* (Koi)	0.25 ± 0.049	0.81 ± 0.17	0.025 ± 0.004	0.78 ± 0.28	[74]
				*Heteropneustes fossilis* (Shing)	0.27 ± 0.059	0.92 ± 0.32	0.016 ± 0.012	0.97 ± 0.26	
				*Colisa fasciata* (Kholisha)	0.18 ± 0.022	0.52 ± 0.30	0.019 ± 0.011	0.70 ± 0.33	
				*Channa striata* (Shoil)	0.25 ± 0.060	0.78 ± 0.27	0.020 ± 0.010	0.69 ± 0.17	
				*Notopterus notopterus* (Foli)	0.25 ± 0.057	0.82 ± 0.36	0.022 ± 0.017	1.1 ± 0.31	
				*Tenualosa ilisha* (Hilsha)	0.51 ± 0.18	0.51 ± 0.47	0.17 ± 0.19	0.48 ± 0.22	
				*Corica soborna* (Kachki)	0.37 ± 0.26	0.58 ± 0.42	0.20 ± 0.20	0.44 ± 0.34	
Standards					1.0	0.5	0.1	1.0	[78]

Notes: NA, not applicable/reported. For metals and metalloids concentrations, some values were with standard errors.

**Table 5:** Heavy metal and metalloid pollution in vegetables and rice (mg/kg).

City	Sampling Site	Sampling Period	Common Name	Scientific Name	Sample No	As	Pb	Cd	Cr	Reference
Dhaka	Surrounding DEPZ	January2005 to February 2006	Egg plant	*Solanum melongena*	12	NA	11.97 (2.17–21.14)	2.91 (0.82–4.85)	6.27 (1.19–11.47)	[92]
			Chilli	*Capsicum annuum* L.	10	NA	13.81 (9.12–18.55)	2.18 (1.27–3.50)	3.70 (2.94–4.61)	
			Tomato	*Solanum lycopersicum*	13	NA	14.15 (7.89–20.54)	2.39 (0.89–3.70)	9.03 (7.67–10.39)	
			Lady’s finger	*Abelmoschus esculentus*	11	NA	15.72 (9.88–24.65)	2.81 (1.03–4.65)	6.64 (2.28–11.84)	
			Cabbage	*Brassica oleracea*	13	NA	22.09 (17.35–26.34)	2.05 (1.05–3.10)	7.58 (6.10–8.74)	
Dhaka	Surrounding Hazaribagh leather industrial area of Dhaka city	NA	Spinach	*Spinacia oleracea*	4	0.26±0.22	11.48 ± 4.98	0.32 ± 0.094	44.48 ± 12.59	[27]
Dhaka	Kawran Bazar (market-based study)	NA	Tomato	*Solanum lycopersicum*	NA	NA	0.00–0.025	0.00–0.001	0.01–0.02	[97]
			Red amaranth	*Amaranthus gangeticus* L.	NA	NA	0.00–0.044	0.00–0.001	NA	
			Rice	*Oryza sativa*	10	0.00–0.70	0.00–0.08	0.003–1.616	0.00–0.01	
Dhaka	Surrounding the Turag river	February–March 2010	Tomato	*Solanum lycopersicum*	6	0.01 ± 0.00	0.23 ± 0.05	0.05 ± 0.01	1.23 ± 0.32	[96]
			Bottle gourd	*Lagenaria siceraria*	6	0.02 ± 0.00	0.69 ± 0.15	0.04 ± 0.01	0.91 ± 0.24	
			Brinjal	*Solanum melongena*	6	0.04 ± 0.01	0.07 ± 0.02	0.24 ± 0.05	1.02 ± 0.27	
			Pumpkin	*Cucurbita maxima*	6	0.02 ± 0.00	0.25 ± 0.06	0.01 ± 0.00	1.45 ± 0.38	
			Green amaranth	*Amaranthus viridis* L.	6	0.19 ± 0.04	0.54 ± 0.56	0.15 ± 0.03	2.28 ± 0.60	
			Red amaranth	*Amaranthus paniculatus* L.	6	0.15 ± 0.03	1.99 ± 0.44	0.84 ± 0.17	2.13 ± 0.56	
			Chilli	*Capsicum annuum* L.	6	0.01 ± 0.00	0.17 ± 0.04	0.33 ± 0.07	1.23 ± 0.32	
			Banana	*Musa* sp.	6	0.01 ± 0.00	0.11 ± 0.02	0.05 ± 0.01	1.27 ± 0.34	
Whole country	Markets		Brinjal	*Solanum melongena*	12	0.006 ± 0.001	0.011 ± 0.011	0.041 ± 0.032	0.497 ± 0.029	[98]
			Tomato	*Solanum lycopersicum*	12	0.006 ± 0.002	0.005 ± 0.004	0.056 ± 0.004	0.795 ± 0.059	
			Potato	*Solanum tuberosum*	12	0.006 ± 0.001	0.007 ± 0.006	0.013 ± 0.007	0.528 ± 0.051	
			Green chili	*Capsicum annuum*	12	0.004 ± 0.001	0.006 ± 0.005	0.023 ± 0.011	0.650 ± 0.039	
			Bean	*Phaseolus vulgaris*	12	0.018 ± 0.007	0.057 ± 0.050	0.008 ± 0.001	1.110 ± 0.054	
			Banana	*Musa acuminata*	12		0.003 ± 0.003		0.317 ± 0.012	
			Carrot	*Daucus carota*	12	0.006 ± 0.001	0.029 ± 0.025	0.023 ± 0.003	0.296 ± 0.021	
Pabna	Pakshi (6)	NA	Potato	*Solanum tuberosum*	NA	<0.1	0.377 ± 0.02	<0.1	<0.1	[37]
			Red amaranth	*Amaranthus cruentus*	NA	<0.1	1.036 ± 0.01	<0.1	<0.1	
			Green amaranth	*Spinach amaranth*	NA	<0.1	1.596 ± 0.01	<0.1	<0.1	
			Carrot	*Daucus carota*	NA	<0.1	0.304 ± 0.01	<0.1	<0.1	
			Tomato	*Solanum lycopersicum*	NA	<0.1	0.161 ± 0.01	<0.1	0.75 ± 0.01	
			Cabbage	*Brassica oleracea*	NA	<0.1	0.119 ± 0.01	<0.1	0.495 ± 0.01	
			Brinjal	*Solanum melongena*	NA	<0.1	0.465 ± 0.01	<0.1	0.436 ± 0.01	
Brahmanbaria	Matlab (household study) 13		Amaranth	*Amaranthus*	13 household	0.0228 ± 0.0037	NA	0.033 ± 0.001	NA	[90]
			Bitter gourd	*Momordica charantia*	13 household	0.0031 ± 0.0026	NA	0.0211± 0.0005	NA	
			Eggplant	*Solanum melongena*	13 household	0.007 ± 0.003	NA	0.027± 0.0018	NA	
Chittagong	Industrial Area (Vatiary)	NA	Water Spinach	*Ipomoea aquatica*	NA	NA	0.73 ± 0.009	NA	3.21 ± 0.023	[93]
			Bottle gourd	*Lagenaria siceraria*	NA	NA	1.16 ± 0.001	NA	0.22 ± 0.008	
Patuakhali	Surrounding the Paira river	August−September 2012	Tomato	*Solanum lycopersicum*	10	0.2 ± 0.5	0.2 ± 0.2	0.07± 0.07	0.6 ± 0.2	[99]
			Potato	*Solanum tuberosum*	10	0.1 ± 0.07	0.4 ± 0.7	0.1± 0.2	0.7 ± 0.4	
			Green amaranth	*Amaranthus hybridus*	10	0.2 ± 0.1	1.2 ± 1.3	0.3± 0.5	1.3 ± 0.7	
			Red amaranth	*Amaranthus gangeticus* L.	10	0.1 ± 0.1	0.9 ± 0.8	0.3 ± 0.3	1.5 ± 1.3	
			Brinjal	*Solanum melongena*	10	0.04 ± 0.04	0.3 ± 0.4	0.1 ± 0.1	0.8 ± 0.4	
			Bottle gourd	*Lagenaria siceraria*	10	0.8 ± 2.5	0.4 ± 0.7	0.1 ± 0.09	0.7 ± 0.3	
			Chili	*Capsicum annuum* L.	10	0.2 ± 0.2	0.2 ± 0.2	0.1 ± 0.1	0.7 ± 0.3	
			Carrot	*Daucus carota*	10	0.1 ± 0.09	0.5 ± 0.7	0.06 ± 0.09	0.8 ± 0.3	
			Onion	*Allium cepa*	10	0.1 ± 0.05	0.4 ± 0.3	0.2 ± 0.3	0.8 ± 0.2	
			Bean	*Phaseolus vulgaris*	10	0.1 ± 0.06	1.0 ± 1.9	0.08 ± 0.1	0.8 ± 0.3	
Gazipur	Surrounding the roadside of Joydebpur	Distance from highway 0 m	Bottle gourd	*Lagenaria siceraria*	NA	3	3.43 ± 0.15	0.18 ± 0.01	NA	[95]
		Distance from highway 100 m	Bottle gourd	*Lagenaria siceraria*	NA	3	2.38 ± 0.13	0.15 ± 0.02	NA	
		Distance from highway 0 m	Pumpkin	*Cucurbita maxima*	NA	3	4.76 ± 1.03	0.20 ± 0.02	NA	
		Distance from highway 100 m	Pumpkin	*Cucurbita maxima*	NA	3	2.13 ± 0.12	0.18 ± 0.01	NA	
Standards						0.1	0.1	0.05	2.3	[91]

Notes: NA, not applicable/reported.

## References

[1] Wang S.W., Shi X.L. (2001). Molecular mechanisms of metal toxicity and carcinogenesis. Mol. Cell Biochem..

[2] Tchounwou P.B., Yedjou C.G., Patlolla A.K., Sutton D.J. (2012). Heavy metal toxicity and the environment. Mol. Clin. Environ. Toxicol..

[3] National Research Council (2001). Arsenic in Drinking Water: 2001 Update.

[4] Bangladesh Population. http://www.worldometers.info/world-population/bangladesh-population/.

[5] Alam M.G.M., Allinson G., Stagnitti F., Tanaka A., Westbrooke M. (2002). Arsenic contamination in Bangladesh groundwater: A major environmental and social disaster. Int. J. Environ. Health Res..

[6] Chakraborti D., Singh S.K., Rahman M.M., Dutta R.N., Mukherjee S.C., Pati S., Kar P.B. (2018). Groundwater Arsenic Contamination in the Ganga River Basin: A Future Health Danger. Int. J. Environ. Res. Public Health.

[7] UNICEF (United Nations International Children’s Emergency Fund) Arsenic Mitigation in Bangladesh.

[8] Ali M.M., Ali M.L., Islam M.S., Rahman M.Z. (2016). Preliminary assessment of heavy metals in water and sediment of Karnaphuli River, Bangladesh. Environ. Nanotech. Moni. Mang..

[9] Banu Z., Chowdhury M.S.A., Hossain M.D., Nakagami K.I. (2013). Contamination and Ecological Risk Assessment of Heavy Metal in the Sediment of Turag River, Bangladesh: An Index Analysis Approach. J. Water Resour. Prot..

[10] Hasan I., Rajia S., Kabir K.A., Latifa G.A. (2009). Comparative Study on the Water Quality Parameters in Two Rural and Urban Rivers Emphasizing on the Pollution Level. Glob. J. Environ. Res..

[11] Zakir H.M., Sharmin S., Shikazono N. (2006). Heavy metal pollution assessment in water and sediments of Turag River at Tongi area in Bangladesh. Int. J. Lakes Rivers.

[12] Ahmed M.K., Baki M.A., Kundu G.K., Islam M.S., Islam M.M., Hossain M.M. (2016). Human health risks from heavy metals in fish of Buriganga river, Bangladesh. Springerplus.

[13] Islam F., Rahman M., Khan S.S.A., Ahmed B., Bakar A., Halder M. (2013). Heavy metals in water, sediment and some fishes of karnofuly river, bangladesh. Int. J. Environ. Res..

[14] Rahman S.H., Khanam D., Adyel T.M., Islam M.S., Ahsan M.A., Akbor M.A. (2012). Assessment of Heavy Metal Contamination of Agricultural Soil around Dhaka Export Processing Zone (DEPZ), Bangladesh: Implication of Seasonal Variation and Indices. Appl. Sci. Basel.

[15] Rakib M.A., Ali M., Akter M.S., Bhuiyan M.A.H. (2014). Assessment of Heavy Metal (Pb, Zn, Cr and Cu) Content in Roadside Dust of Dhaka Metropolitan City, Bangladesh. Int. Res. J. Environ. Sci..

[16] Brevik E.C., Burgess L.C. (2012). Soils and Human Health.

[17] Kabir E., Ray S., Kim K.H., Yoon H.O., Jeon E.C., Kim Y.S., Cho Y.S., Yun S.T., Brown R.J.C. (2012). Current Status of Trace Metal Pollution in Soils Affected by Industrial Activities. Sci. World J..

[18] Islam M.A., Romic D., Akber M.A., Romic M. (2018). Trace metals accumulation in soil irrigated with pollutedwater and assessment of human health risk from vegetable consumption in Bangladesh. Environ. Geochem. Health.

[19] Chen Y., Wang C.X., Wang Z.J. (2005). Residues and source identification of persistent organic pollutants in farmland soils irrigated by effluents from biological treatment plants. Environ. Int..

[20] Fergusson J.E. (1991). The Heavy Elements: Chemistry, Environmental Impact and Health Effects.

[21] Meharg A.A., Rahman M. (2003). Arsenic contamination of Bangladesh paddy field soils: Implications for rice contribution to arsenic consumption. Environ. Sci. Technol..

[22] Duxbury J.M., Mayer A.B., Lauren J.G., Hassan N. (2003). Food chain aspects of arsenic contamination in Bangladesh: Effects on quality and productivity of rice. J. Environ. Sci. Health A Tox. Hazard Subst. Environ. Eng..

[23] Ratul A.K., Hassan M., Uddin M.K., Sultana M.S., Akbor M.A., Ahsan M.A. (2018). Potential health risk of heavy metals accumulation in vegetables irrigated with polluted river water. Int. Food Res. J..

[24] Ahsan D.A., Valls T.A.D. (2011). Impact of arsenic contaminated irrigation water in food chain: An overview from Bangladesh. Int. J. Environ. Res..

[25] Joseph T., Dubey B., Mcbean E.A. (2015). A critical review of arsenic exposures for Bangladeshi adults. Sci. Total Environ..

[26] Raessler M. (2018). The Arsenic Contamination of Drinking and Groundwaters in Bangladesh: Featuring Biogeochemical Aspects and Implications on Public Health. Arch. Environ. Con. Tox..

[27] Mottalib M.A., Somoal S.H., Aftab M., Shaikh A., Islam M.S. (2016). Heavy metal concentrations in contaminated soil and vegetables of tannery area in Dhaka, Bangladesh. Int. J. Curr. Res..

[28] Zakir H.M., Sultana N., Akter M. (2014). Heavy metal contamination in roadside soils and grasses: A case study from Dhaka city, Bangladesh. J. Chem. Biol. Phys. Sci..

[29] Zakir H.M., Sumi S.A., Sharmin S., Mohiuddin K.M., Kaysar S. (2015). Heavy metal contamination in surface soils of some industrial areas of Gazipur, Bangladesh. J. Chem. Biol. Phys. Sci..

[30] Begum K., Mohiuddin K.M., Zakir H.M., Rahman M., Hasan M.N. (2014). Heavy Metal Pollution and Major Nutrient Elements Assessment in the Soils of Bogra City in Bangladesh. Can. Chem. Trans..

[31] Alamgir M., Islam M., Hossain N., Kibria M.G., Rahman M.M. (2015). Assessment of Heavy Metal Contamination in Urban Soils of Chittagong City, Bangladesh. Int. J. Plant. Soil Sci..

[32] Begum M., Huq S.I. (2016). Heavy metal contents in soils affected by industrial activities in a southern district of Bangladesh. Bangladesh J. Sci. Res..

[33] Rahman M.T., Ziku A.L.M.E., Choudhury T.R., Ahmed J.U., Mottaleb M.A. (2015). Heavy metal contaminations in vegetables, soils and river water: A comprehensive study of Chilmari, Kurigram, Bangladesh. Int. J. Environ. Ecol. Fam. Urban Stud..

[34] Proshad P., Islam M.S., Kormoker T. (2018). Assessment of heavy metals with ecological risk of soils in the industrial vicinity of Tangail district, Bangladesh. Int. J. Adv. Geosci..

[35] Halim M.A., Majumder R.K., Zaman M.N. (2015). Paddy soil heavy metal contamination and uptake in rice plants from the adjacent area of Barapukuria coal mine, northwest Bangladesh. Arab. J. Geosci.

[36] Bhuiyan M.A.H., Parvez L., Islam M.A., Dampare S.B., Suzuki S. (2010). Heavy metal pollution of coal mine-affected agricultural soils in the northern part of Bangladesh. J. Hazard. Mater..

[37] Tasrina R.C., Rowshon A. (2015). Heavy Metals Contamination in Vegetables and its Growing Soil. Int. J. Environ. Anal. Chem..

[38] USEPA (United States Environmental Protection Agency) (2002). Supplemental Guidance for Developing Soil Screening Levels for Superfund Sites.

[39] Department of Environment (1997). Environment Conservation Rules.

[40] Hasnine M.T. (2017). Heavy Metal Contamination in Agricultural Soil at DEPZA, Bangladesh. Environ. Ecol. Res..

[41] Kibria G., Hossain M.M., Mallick D., Lau T.C., Wu R. (2016). Monitoring of metal pollution in waterways across Bangladesh and ecological and public health implications of pollution. Chemosphere.

[42] Harper C., Lllados F., Sage G., Colman J., Chappel L., Ingermann L., Odin M., Osier M., Chou S. (2007). Toxicological profile for arsenic.

[43] McArthur J.M., Ghosal U., Sikdar P.K., Ball J.D. (2016). Arsenic in Groundwater: The Deep Late Pleistocene Aquifers of the Western Bengal Basin. Environ. Sci. Technol..

[44] Smith A.H., Lingas E.O., Rahman M. (2000). Contamination of drinking-water by arsenic in Bangladesh: A public health emergency. Bull World Health Organ..

[45] McCarty K., Hanh H., Kim K. (2011). Arsenic geochemistry and human health in South East Asia. Rev. Environ. Health.

[46] Sawyer C.N., Mccarty P.L., Parkin G.F. (2003). Chemistry for Environmental and Engineering and Science.

[47] Chakraborti D., Rahman M.M., Mukherjee A., Alauddin M., Hassan M., Dutta R.N., Pati S., Mukherjee S.C., Roy S., Quamruzzman Q. (2015). Groundwater arsenic contamination in Bangladesh-21 Years of research. J. Trace Elem. Med. Biol..

[48] BGS-DPHE (1999). Groundwater Studies for Arsenic Contamination in Bangladesh. Final Report.

[49] Chowdhury M.A.I., Uddin M.T., Ahmed M.F., Ali M.A. (2006). How Does Arsenic Contamination of Groundwater Causes Severity and Health Hazard in Bangladesh?. J. Appl. Sci.

[50] Flanagan S.V., Johnston R.B., Zheng Y. (2012). Arsenic in tube well water in Bangladesh: Health and economic impacts and implications for arsenic mitigation. Bull World Health Organ..

[51] Davis C. (2003). Chapter 32—Arsenic mitigation in Bangladesh: Progress of the UNICEF-DPHE Arsenic Mitigation Project 2002. Arsenic Exposure & Health Effects V.

[52] Milton A.H., Hore S.K., Hossain M.Z., Rahman M. (2012). Bangladesh arsenic mitigation programs: Lessons from the past. Emerg. Health Threats J..

[53] Khan M.M., Aklimunnessa K., Kabir M., Mori M. (2007). Determinants of drinking arsenic-contaminated tubewell water in Bangladesh. Health Policy Plan..

[54] Myers S.S., Gaffikin L., Golden C.D., Ostfeld R.S., Redford K.H., Ricketts T.H., Turner W.R., Osofsky S.A. (2013). Human health impacts of ecosystem alteration. Proc. Natl. Acad. Sci. USA.

[55] Aktar P., Moonajilin M.S. (2017). Assessment of Water Quality Status of Turag River Due to Industrial Effluent. Int. J. Eng. Inf. Syst..

[56] Brady T. (2014). Working themselves to death, the Bangladesh men and women tanning leather for a pittance in one of the world’s top 30 most polluted locations. Daily Mail.

[57] FAO Water Pollution from Agriculture: A Global Review. www.fao.org/3/a-i7754e.pdf.

[58] Uddin M.J., Khanom S., Mamun S.A., Parveen Z. (2016). Effects of irrigation water on some vegetables around industrial areas of Dhaka. Bangladesh J. Sci. Res..

[59] Department of Environment (2015). River water quality report, 2014.

[60] Mohiuddin K.M., Alam M.M., Ahmed I., Chowdhury A.K. (2016). Heavy metal pollution load in sediment samples of the Buriganga river in Bangladesh. J. Bangladesh Agril. Univ..

[61] Saha P.K., Hossain M.D. Assessment of Heavy Metal Contamination and Sediment Quality in the Buriganga River, Bangladesh. Proceedings of the 2nd International Conference on Environmental Science and Technology.

[62] Mohiuddin K.M., Islam M.S., Basak S., Abdullah H.M., Ahmed I. (2016). Status of heavy metal in sediments of the Turag river in Bangladesh. Progress. Agric..

[63] Ahmad M.K., Islam S., Rahman S., Haque M.R., Islam M.M. (2010). Heavy Metals in Water, Sediment and Some Fishes of Buriganga River, Bangladesh. Int. J. Environ. Res..

[64] Islam S.M.D., Bhuiyan M.A.H., Rume T., Mohinuzzaman M. (2016). Assessing Heavy Metal Contamination in the Bottom Sediments of Shitalakhya River, Bangladesh; Using Pollution Evaluation Indices and Geo-spatial Analysis. Pollut. Res..

[65] Sarkar M., Rahman A.K.M.L., Islam J.B., Ahmed K.S., Uddin M.N., Bhoumik N.C. (2015). Study of hydrochemistry and pollution status of the Buriganga river, Bangladesh. Bangladesh J. Sci. Ind. Res..

[66] Bhuiyan M.A., Dampare S.B., Islam M.A., Suzuki S. (2015). Source apportionment and pollution evaluation of heavy metals in water and sediments of Buriganga River, Bangladesh, using multivariate analysis and pollution evaluation indices. Environ. Monit Assess..

[67] Mokaddes M.A.A., Nahar B.S., Baten M.A. (2012). Status of Heavy Metal Contaminations of Lake Water of Dhaka Metropolitan City. J. Environ. Sci. Nat. Resour..

[68] Islam M.S., Ahmed M.K., Raknuzzaman M., Habibullah-Al-Mamun M., Islam M.K. (2015). Heavy metal pollution in surface water and sediment: A preliminary assessment of an urban river in a developing country. Ecol. Indic..

[69] Shil S.C., Islam M.S., Irin A., Tusher T.R., Hoq M.E. (2017). Heavy Metal Contamination in Water and Sediments of Passur River near the Sundarbans Mangrove of Bangladesh. J. Environ. Sci. Nat. Resour..

[70] Ali M.M., Ali M.L., Islam M.S., Rahman M.Z. (2018). Assessment of toxic metals in water and sediment of Pasur River in Bangladesh. Water Sci. Technol..

[71] Pescod M.B. (1992). Wastewater Treatment and Use in Agriculture.

[72] Mohiuddin K.M., Ogawa Y., Zakir H.M., Otomo K., Shikazono N. (2011). Heavy metals contamination in water and sediments of an urban river in a developing country. Int. J. Environ. Sci. Technol..

[73] Zakir H.M., Rahman M.M., Rahman A., Ahmed I., Hossain M.A. (2013). Heavy Metals and Major Ionic Pollution Assessment in Waters of Midstream of the River Karatoa in Bangladesh. J. Environ. Sci. Nat. Resour..

[74] Islam M.S., Habibullahalmamun M. (2017). Accumulation of trace elements in sediment and fish species of Paira River, Bangladesh. AIMS Environ. Sci..

[75] Rudnick R.L., Gao S. (2014). 4.1—Composition of the Continental Crust. Am. Miner..

[76] USEPA (1999). Screening Level Ecological Risk Assessment Protocol for Hazardous Waste Combustion Facilities. EPA530-D-99-001C.

[77] Bhuyan M.S., Bakar M.A., Akhtar A., Islam M.S. (2016). Heavy Metals Status in Some Commercially Important Fishes of Meghna River Adjacent to Narsingdi District, Bangladesh: Health Risk Assessment. Am. J. Life Sci..

[78] WHO (2002). Codex Alimentarius—General Standards for Contaminants and Toxins in Food. Reference CX/FAC 02/16.

[79] Dey S., Das J., Manchur M.A. (2015). Studies on Heavy Metal Pollution of Karnaphuli River, Chittagong, Bangladesh. J. Environ. Sci. Toxicol. Food Technol..

[80] Siddiquee N.A., Parween S., Quddus M.M.A., Barua P. (2009). Heavy Metal Pollution in Sediments at Ship Breaking Area of Bangladesh. Asian J. Water Environ. Pollut..

[81] Samad M.A., Mahmud Y., Adhikary R.K., Rahman S.B.M., Haq M.S., Rashid H. (2015). Chemical Profile and Heavy Metal Concentration in Water and Freshwater Species of Rupsha River, Bangladesh. Am. J. Environ. Prot..

[82] Sabbir W., Rahman M.Z., Hasan M.M., Khan M.N., Ray S. (2018). Assessment of heavy metals in river water, sediment and fish mussel in rupsha river under Khulna district, Bangladesh. Int. J. Expt. Agric..

[83] Hasan S.J., Tanu M.B., Haida M.I., Ahmed T., Rubel A.S. (2015). Physico-chemical characteristics and accumulation of heavy metals in water and sediments of the river Dakatia, Bangladesh. Int. J. Fish. Aquat. Stud..

[84] Alam M.G.M., Snow E.T., Tanaka A. (2003). Arsenic and heavy metal contamination of vegetables grown in Samta village, Bangladesh. Sci. Total Environ..

[85] Mingorance M.D., Valdés B., Oliva S.R. (2007). Strategies of heavy metal uptake by plants growing under industrial emissions. Environ. Int..

[86] Chang C.Y., Yu H.Y., Chen J.J., Li F.B., Zhang H.H., Liu C.P. (2014). Accumulation of heavy metals in leaf vegetables from agricultural soils and associated potential health risks in the Pearl River Delta, South China. Environ. Monit Assess..

[87] Meharg A.A., Williams P.N., Adomako E., Lawgali Y.Y., Deacon C., Villada A., Cambell R.C.J., Sun G., Zhu Y.G., Feldmann J. (2009). Geographical Variation in Total and Inorganic Arsenic Content of Polished (White) Rice. Environ. Sci. Technol..

[88] Rahman M.A., Hasegawa H. (2011). High levels of inorganic arsenic in rice in areas where arsenic-contaminated water is used for irrigation and cooking. Sci. Total. Environ..

[89] Al Rmalli S.W., Haris P.I., Harrington C.F., Ayub M. (2005). A survey of arsenic in foodstuffs on sale in the United Kingdom and imported from Bangladesh. Sci. Total Environ..

[90] Khan S.I., Ahmed A.K.M., Yunus M., Rahman M., Hore S.K., Vahter M., Wahed M.A. (2010). Arsenic and cadmium in food-chain in Bangladesh—An exploratory study. J. Health Popul. Nutr..

[91] FAO/WHO (2011). Food Standards Programme on Contaminants in Foods. CF/5 INF/1.

[92] Ahmad J.U., Goni M.A. (2010). Heavy metal contamination in water, soil, and vegetables of the industrial areas in Dhaka, Bangladesh. Environ. Monit. Assess..

[93] Parvin R., Sultana A., Zahid M.A. (2014). Detection of Heavy Metals in Vegetables Cultivated In Different Locations in Chittagong, Bangladesh. IOSR-JESTFT.

[94] Islam M.S., Ahmed M.K., Habibullah-Al-Mamun M., Raknuzzaman M., Ali M.M., Eaton D.W. (2016). Health risk assessment due to heavy metal exposure from commonly consumed fish and vegetables. Environ. Syst. Decis..

[95] Naser H.M., Sultana S., Gomes R., Noor S. (2012). Heavy metal pollution of soil and vegetable grown near roadside at Gazipur. Bangladesh J. Agric. Res..

[96] Islam M.S., Hoque M.F. (2014). Concentrations of heavy metals in vegetables around the industrial area of Dhaka city, Bangladesh and health risk assessment. Int. Food Res. J..

[97] Mih R., Azam H.M., Majed N. (2017). Consumption of heavy metal contaminated foods and associated risks in Bangladesh. Environ. Monit. Assess..

[98] Shaheen N., Irfan N.M., Khan I.N., Islam S., Islam M.S., Ahmed M.K. (2016). Presence of heavy metals in fruits and vegetables: Health risk implications in Bangladesh. Chemosphere.

[99] Islam M.S., Ahmed M.K., Habibullah-Al-Mamun M. (2015). Determination of Heavy Metals in Fish and Vegetables in Bangladesh and Health Implications. Hum. Ecol. Risk Assess..

